# Supplementary material for: Strategies for primary HPV test-based cervical cancer screening programme in resource-limited settings in India: Results from a quasi-experimental pragmatic implementation trial
Source: PLoS One. 2024 Apr 5;19(4):e0301385. doi: 10.1371/journal.pone.0301385 (PMC10997089; doi:10.1371/journal.pone.0301385)
Supplement: S1 File — (DOCX) [file pone.0301385.s002.docx]

# Codes obtained using inductive coding of interviews with project managers (tribal T, urban U, rural R, lab manager L, Data manager D)

1. Role, in charge tribal site and program manager for tribal (T)
2. Develop KAP tool, train for survey, train urban site (U)
3. Train how to gather and educate the community (U) evening best time to give mass education (U) shift duties for nurses needed (U) (only in the late evening women available and distance 2 h away (T) had to change to evening for rural (R)
4. Develop health education material with health educator, poster, and video (U) video developed to improve education (R)
5. Sensitive topic so women hesitant for sessions (R) rainy season urban hesitant to come (R)
6. Train nurses how to facilitate self-sampling (U)
7. Train to maintain a register (U)
8. Train to counsel positive women, observation in field (U)
9. Obtaining permissions, identify and arranging venue, organise education sessions (U, T) chose common areas (R)
10. Ensuring correct messages (U)
11. Monitoring visits weekly (U)
12. Help in mass education (U)
13. Kit distribution (U, T) getting them back, ensuring follow up (T) maintained a note (R)
14. Allocate responsibilities, manage staff, hire field staff (T)
15. Coordinate, scheduling, planning (R)
16. Review meetings once in 2 weeks with number screened and challenges (R)
17. Need one program manager per site (R) if in PHC make a schedule for the PHCs
18. Senior Programmer, previously managed VIA program, data management (D)
19. create Access form, data entry, checking, adding HPV results to data, analysis, (D)
20. Kept forms and consent forms secure (D)
21. Role of processing, giving kits and collecting, reporting, Empty kits stored in lab (L)
22. Created ID numbers for each site (L)
23. Checked form and tube numbers, checked for medium presence (L)
24. Challenges less than with VIA (T)
25. Women open to doing the self-sampling although we were apprehensive (T) women had no previous experience with screening unlike rural areas (T) Women asking for kits (U) called it ‘kutchi’ test (R), message spread among women (R)
26. Support from local NGO (T)
27. Govt nurses appreciated free testing (R)
28. What was challenging was reaching the women, used various sources to gather women (T)
29. Not sure if screening invitation reached all (T), working women working from 8 to night in shoe companies or tailoring (U) – would have missed them; (R) evening education done (T, R)
30. Health workers had old census data which needed time to update (R)
31. Worried about injury with brush (U)
32. Women asymptomatic, not interested in follow up (T)
33. Vellore district - not used to health care unless sick.
34. Special camps arranged in Vellore
35. Sent vehicle to homes, even then some women did not feel they need it
36. Nurses hesitant to counsel positive women at their homes because of crowded homes/no privacy (U)
37. Some women missed special camps and were referred by health worker (periods, gone out) (T) some women missed follow up because of periods (U)
38. 3-4 women came to monthly once gynae clinic in tribal site 1 hour away, went away because made to wait.
39. Seasonal migration - follow up issue.
40. Realised negative people not getting results so told health workers to inform (T)
41. Half had phones but no coverage; could not use phone for follow up (T); in rural if not reachable by phone made home visits (R) urban and rural home visits (R)
42. Facilitators: Team, nurses, doctors (T); manpower and rapport, urban PHC and CMC site in urban, health education, training coordinators, nurses (U)
43. Facilitators: good posters and videos (R)
44. Facilitator: Health workers are tribal women (T)
45. Relationship with communities (T)/knowing the study site (U)
46. All facilities for screening and follow up (T), our strategy of health education, screening, diagnosis, and confirmation (U)
47. Coverage was good for a program (U) good for tribal settings (T)
48. RUHSA experience with VIA since 2008-09 (D) regularly compiling data (D)
49. Issues of visibility of videos in large groups (T)
50. No infrastructure changes needed in lab (L), difficult but adjusted, small space enough.
51. Two training classes (lab). 6 hours per session with company lab person. First session taught me, next supervised performance (L)
52. Person with diploma in medical lab technology training can do the HPV test (L) Health workers cannot do
53. Test will give report whatever the sample volume, as positive or negative by showing colours (L)
54. Control runs with each batch.
55. Stored samples in fridge till 90 samples (L)
56. 90 samples processing take 4 to 6 hours of lab tech time; can process only 90 samples at a time is a major problem (L). procedure should be with less number of samples and less time.
57. Needed to repeat samples if full of blood, chemical spilt or brush missing (L)
58. One failure of results so called company who replaced control reagents (L)

# Themes and Categories under each theme

Challenges and solutions for:

1. **Identification and invitation of eligible women (codes 28-30, 51)**

- Physically reaching tribal women to invite
- Need to update existing census to identify target group.
- Need for evening programs.
- Timing for group meetings

1. **Screening test and laboratory issues (code 24, 31, 50-58)**

- Less challenging than VIA
- Worries in women about injury
- Staff who can do the lab test.
- Storage of samples
- Time taken per run.
- Waiting for 90 samples
- Quality issues of some samples

1. **Follow-up challenges related to target population (codes 31-33, 39)**

Asymptomatic women not interested in all sites

Tribal:

- Vellore district tribal women not used to health care seeking unless very sick
- Some women missed due to periods or migrating for work
- Phone coverage poor in tribal area

1. **Follow up challenges related to health system (codes 34-36, 38, 40-49)**

- Tribal women waiting long for follow up tests; staff counselled
- Focused on screen positive women
- Not sure if tribal health workers informed negatives
- Protocol deviation for follow up
- Monthly once tribal gynae clinic
- Distance, sent vehicle to pick up tribal women
- Long waiting time for colposcopy
- Special camps held for tribal
