# Supplementary material for: Strategies for primary HPV test-based cervical cancer screening programme in resource-limited settings in India: Results from a quasi-experimental pragmatic implementation trial
Source: PLoS One. 2024 Apr 5;19(4):e0301385. doi: 10.1371/journal.pone.0301385 (PMC10997089; doi:10.1371/journal.pone.0301385)
Supplement: Attachment — Submitted filename: Response to Reviewers.docx [file pone.0301385.s003.docx]

**Response to Reviewers**

On behalf of all the authors, I want to place on record our sincere thanks to the reviewers for an excellent review and appreciation of our hard work and study methods chosen. We see the reviewers have a good understanding of the implementation science research. We want to thank the editors too for identifying the right people for the review.

**Response to academic editor’s review**

- We haven’t made any changes in the financial disclosure.
- We do not have any figure in the manuscript.
- We have used publicly available, routinely used laboratory tests for this research, so depositing the laboratory protocols in protocol.io is not applicable.
- We have followed the PLOS ONE's style requirements while formatting to the best of our ability
- We have removed the reference no.21 from the previous submitted version as it is not a published article. It is a reference to data from the existing health information system of the research organisation. We have mentioned that in parenthesis in the text.

21. Health information system, RUHSA. Christian Medical College Vellore.

- We have edited the references 4 and 5 to comply with PLOS ONE publication criteria

**Reviewer’s comments**

Reviewer 1#

We would like to thank the reviewer once again for the excellent review and appreciation of our work and study methods.

Reviewer 2#
**1. The study identifies several limitations. Firstly, its quasi-experimental design limits the ability to establish causality.**

As the reviewer has suggested, we have added a few additional lines to explain why a quasi-experimental design was chosen and its merits in 342 to 355 lines.

**2. Secondly, self-reported data may introduce recall bias and affect the reliability of some findings.**

We have mentioned in the methodology section that the study focused on rigorous training of the data collectors to reduce the recall bias.

Also the intervention was rolled out only for a short period of 8 months and therefore pre-post intervention qualitative data collected on the barriers and challenges of cancer screening may not be significantly affected by recall bias.

As the main outcomes we measured were screening and follow-up rates which were objective measures based on our program data, these are not affected by recall bias.

**3. Thirdly, the study focuses on specific geographic areas in India, which may limit the generalizability of the results to other settings.**

Across all states in India, the society has been broadly divided into tribal, rural and urban societies on the basis of their geographical surroundings and socio-cultural characteristics. We have chosen the 3 resource limited settings within the existing geographic divisions. Though there may be some sociocultural differences in the uptake of interventions across states, the 3 sites largely represent rural, tribal and urban slums, the major geographical divisions in India

**4. Additionally, the study does not fully explore the long-term outcomes of the screening program,**

This study is a short term pragmatic pilot trial. There is a fund approved RCT being planned in the study site which will also measure long term outcomes.

**5. the potential impact of socio-cultural factors on screening behaviours is not thoroughly examined.**

We believe the differences that this study have picked up in 3 diverse societies explain the impact of sociocultural factors. We have also examined these through qualitative studies in the area before and after the intervention, which will be published separately.

**6. Finally, the study's reliance on existing health infrastructure might overlook the challenges in settings with more limited resources.**

We agree with the reviewer that the existing health infrastructure in the study sites would have certainly impacted the outcomes. It will be more challenging to implement this screening programme in more limited settings.

**7. Adding these items to the limitations section of the manuscript would make it more informative for the audience.**

As per the reviewer’s suggestion, we have added a few lines under limitations. Please see the lines 342 to 355.

Thank you,

Prof. Rita Isaac

Corresponding Author
